# “Awake” intraoperative functional MRI (ai-fMRI) for mapping the eloquent cortex: Is it possible in awake craniotomy?^☆^

**DOI:** 10.1016/j.nicl.2012.12.002

**Published:** 2012-12-12

**Authors:** Jun-Feng Lu, Han Zhang, Jin-Song Wu, Cheng-Jun Yao, Dong-Xiao Zhuang, Tian-Ming Qiu, Wen-Bin Jia, Ying Mao, Liang-Fu Zhou

**Affiliations:** a Department of Neurosurgery, Huashan Hospital, Fudan University, Shanghai, 200040, China; b Center for Cognition and Brain Disorders, Hangzhou Normal University, Hangzhou, 310015, China

**Keywords:** Functional magnetic resonance imaging (fMRI), Intraoperative magnetic resonance imaging, Tumor, Awake surgery, Intraoperative functional mapping, Surgical planning

## Abstract

As a promising noninvasive imaging technique, functional MRI (fMRI) has been extensively adopted as a functional localization procedure for surgical planning. However, the information provided by preoperative fMRI (pre-fMRI) is hampered by the brain deformation that is secondary to surgical procedures. Therefore, intraoperative fMRI (i-fMRI) becomes a potential alternative that can compensate for brain shifts by updating the functional localization information during craniotomy. However, previous i-fMRI studies required that patients be under general anesthesia, preventing the wider application of such a technique as the patients cannot perform tasks unless they are awake. In this study, we propose a new technique that combines awake surgery and i-fMRI, named “awake” i-fMRI (ai-fMRI). We introduced ai-fMRI to the real-time localization of sensorimotor areas during awake craniotomy in seven patients. The results showed that ai-fMRI could successfully detect activations in the bilateral primary sensorimotor areas and supplementary motor areas for all patients, indicating the feasibility of this technique in eloquent area localization. The reliability of ai-fMRI was further validated using intraoperative stimulation mapping (ISM) in two of the seven patients. Comparisons between the pre-fMRI-derived localization result and the ai-fMRI derived result showed that the former was subject to a heavy brain shift and led to incorrect localization, while the latter solved that problem. Additionally, the approaches for the acquisition and processing of the ai-fMRI data were fully illustrated and described. Some practical issues on employing ai-fMRI in awake craniotomy were systemically discussed, and guidelines were provided.

## Highlights

► The concept of awake intraoperative fMRI (ai-fMRI) was first proposed. ► ai-fMRI is feasible and applicable to detect real-time eloquent areas. ► ai-fMRI is reliable in mapping the sensorimotor cortex. ► The pipeline of ai-fMRI mapping is systematically demonstrated. ► The utilization of ai-fMRI extends the application of fMRI and iMRI.

## 1 Introduction

In surgical neurooncology, the goal of tumor surgery is to maximize tumor resection while sparing important areas of the brain and minimizing the risk of inducing permanent neurological deficits—thus ensuring the patients' quality of life, which is regarded as the highest priority (Brem et al., 2011; Wen and Kesari, 2008). For this reason, neuronavigation based on preoperative functional magnetic resonance imaging (pre-fMRI) is the most common noninvasive tool that can provide additional information concerning the anatomical relationship between the borders of the tumor (especially infiltrative glioma) and eloquent areas (Hirsch et al., 2000; Nimsky et al., 1999, 2004b; Stapleton et al., 1997). This technique uses preoperatively acquired task-related fMRI data to detect activated areas that should be protected during surgery and feeds this information into an image-guided navigational system.

Despite FDA approval and wide usage, pre-fMRI has a major shortcoming: it does not account for the brain deformation or “brain shift” that is secondary to surgical procedures, which is caused by the brain collapsing under the force of gravity to fill the space previously occupied by cerebrospinal fluid and the resected tumor (Nabavi et al., 2001; Nimsky et al., 2000, 2001). The literature has shown that, even after the skull is opened and before any interventional procedure is started, the brain shift could be up to 20 mm (Dorward et al., 1998; Hartkens et al., 2003; Hill et al., 1998; Maurer et al., 1998; Nabavi et al., 2001; Roberts et al., 1998). Because a safety distance between the tumor border and the nearest pre-fMRI activation was suggested to be at least 10 mm (Haberg et al., 2004), the potential brain deformation may cause the pre-fMRI-defined eloquent areas to fall within the dangerous zone. Such inaccuracy in the navigation system will thus lead to serious consequences, such as paralysis or aphasia.

To overcome such a disadvantage of pre-fMRI, the concept of intraoperative fMRI (i-fMRI) was introduced (Gering and Weber, 1998). i-fMRI provides the location information of the eloquent areas during craniotomy and updates real-time data for neuronavigation (Kuhnt et al., 2012), thus helping neurosurgeons visualize the real-time relationship between the tumor and the neighboring functional areas after opening the dura, partially resecting the tumor, or help those facing problems while planning extensive resection. Gasser et al. (2005) first demonstrated the feasibility of i-fMRI in real clinical cases. They successfully localized the somatosensory cortex by stimulating the median and tibial nerves in patients with opened skull. However, this technique is limited in identifying the motor, language or other cognitive areas because of the lack of cooperation from patients caused by general anesthesia, thus restricting its extensive and wide application. Performing awake anesthesia while administrating i-fMRI is required to solve this problem. In recent years, after overcoming technical problems in airway management and draping, our group (Lu et al., 2011) and other neurosurgical centers (Leuthardt et al., 2011; Nabavi et al., 2009; Parney et al., 2010; Weingarten et al., 2009) spearheaded the combination of awake anesthesia and intraoperative structural MRI. These studies demonstrate the possibility of “awake” intraoperative fMRI (ai-fMRI), even though awake anesthesia was only used to perform intraoperative stimulation mapping (ISM) while intraoperative structural MRI (i-sMRI) was used to update anatomical information. The ai-fMRI technique provides a unique opportunity to employ i-fMRI in awake patients, who can then conduct tasks required in surgical procedures. Moreover, ai-fMRI can avoid the notorious problems of ISM, which has the potential of inducing epilepsy and extensive cortical exposure. Therefore, it is urgently required to establish a clinically feasible and widely accepted procedure that describes how to conduct i-fMRI in awake patients.

Here, for the first time, we raise the concept of the ai-fMRI for the localization of real-time eloquent areas during craniotomy. Based on our two years of experience in combining awake surgery and intraoperative MRI, we would like to address several key issues with respect to the clinical realization of ai-fMRI, including 1) performing awake anesthesia in the intraoperative MRI environment, 2) acquiring ai-fMRI data with specific hardware, and 3) processing the ai-fMRI data to reveal task-related areas. We aim to demonstrate the feasibility and reliability of ai-fMRI and provide a firsthand comprehensive document that describes our experience in patient screening, awake anesthesia, ai-fMRI data acquisition and processing, and result comprehension.

## 2 Materials and methods

### 2.1 Patients

For this study, patients were selected who fulfilled the following criteria: 1) cerebral gliomas were preoperatively suspected, and 2) patients with tumors located in motor or language areas as indicated by preoperative MRI. The exclusion criteria consisted of 1) patients with contraindications to MRI scanning or awake craniotomy (i.e., patients with pacemakers, obstructive sleep apnea or severe intracranial hypertension); 2) patients whose pathological diagnoses were not gliomas; and 3) patients with severe cognitive disorders, as evaluated with the Mini Mental State Examination (MMSE) (Folstein et al., 1975), with scores less than 23 (Nabavi et al., 2009). Eleven patients (mean age of 40 ± 10, range from 26 to 58 years, 7 males and 4 females) with gliomas involving the eloquent areas were enrolled in this study. For ten of the 11 patients, the tumors were located in the left cerebral hemisphere, and the other one was located in the right hemisphere. For nine patients, the tumors were adjacent to language areas, while the other two (patients 7 and 11) had tumors near the motor cortex. All patients were tested to be right-handed using the Edinburgh handedness inventory (Oldfield, 1971). The motor functions were normal before operations in all patients. The Huashan Institutional Review Board approved this study. All the patients provided written informed consent prior to the surgeries.

### 2.2 Awake anesthesia

Over the past ten years, awake anesthesia has become an increasingly frequent procedure (Piccioni and Fanzio, 2008). As compared to general anesthesia, it allows patients to cooperate with some experimental tasks for ISM, thus reducing the risk of the operation under general anesthesia (e.g., neurological deficits). In the current work, awake anesthesia was conducted to accomplish ai-fMRI. This is a novel attempt (previously it was only conducted to accomplish ISM). Modern awake anesthetic approaches can be generally divided into two types: monitored anesthesia care (MAC) and asleep–awake–asleep technique (AAA) (Piccioni and Fanzio, 2008). Only MAC protocol is suitable for ai-fMRI because no laryngeal mask airway or endotracheal tubing was applied in the MAC protocol. The biggest advantage of MAC is that the patient could be awakened throughout the operation procedure when needed without intubation and extubation.

The detailed awake anesthesia procedures contained the following steps. First, the patients routinely received intravenous administration of 0.02–0.03 mg/kg midazolam and 5 mg tropisetron for premedication in our hospital. Second, local anesthetic blocks of six nerves on both sides of the scalp are performed using a mixture of lidocaine (0.67%) and ropivacaine (0.5%). Third, the patient was brought into moderate sedation with boluses of intravenous propofol. After that, the head was fixed into its fitted position (supine position, the head were tilted approximately 15–60° to the hemisphere contralateral to the tumor) using a custom-designed high-field MRI-safe head holder (DORO Radiolucent Headrest System, Pro Med Instruments GmbH, Germany). It was important to ensure that the patient was as comfortable as possible, as even slight discomfort may result in total noncompliance during the surgical procedure and MRI scan. During the operation, a combination of remifentanil and propofol was applied for sedation. Next, a craniotomy was routinely performed. Propofol was discontinued before the dura was opened and the patients could be awakened in about 10 min. After completion of ISM and ai-fMRI, the patients were sedated, and the tumor was resected until the neurosurgeons considered that the “maximal safe resection” had been achieved with visual inspection or navigation. Subsequently, sedation was discontinued again (the effect-site concentrations of propofol before discontinuation were shown in Table 1). Only a low dose of remifentanil (Table 1) was infused to maintain the baseline analgesia. The patients were awakened and asked for cooperating with tasks for another ai-fMRI. Although the awake anesthesia for ISM and that for ai-fMRI are similar, there is a major difference between them. That is, a unique draping technique (described in our previous paper, see Lu et al., 2011) was adopted in the current work for ai-fMRI. However, for awake anesthesia for ISM, we usually don't need this unique draping technique (for more details, please see Layout of intraoperative MRI).

### 2.3 Layout of intraoperative MRI

**Fig. 1:**
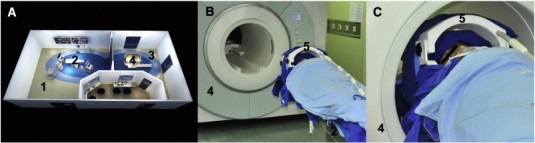
*The layout of the intraoperative MRI and the unique techniques for i-fMRI on awake patients with opened skulls.* (A) Schematic layout of intraoperative MRI, which depicts the operating room (1) with a fixed operating table (2) and the diagnostic room (3) with a movable magnet (4). (B) The magnet (4) is approaching the patient, whose head is covered by two-piece designed coils (5). (C) The minimal draping technique that meets the requirements of both asepsis and awake anesthesia.

The intraoperative MR imaging facility, which was equipped in an intraoperative MRI-integrated neurosurgical suite (IMRIS, Winnipeg, Canada), was designed with a twin-room concept. The core equipment was a ceiling-mounted, movable 3.0 T magnet (MAGNETOM Verio 3.0 T, Siemens AG, Erlangen, Germany) with a 70-cm working aperture. The operating room (OR) is situated adjacent to the diagnostic room (DR) that houses the magnet (Fig. 1A). A wide door connects the two rooms, both of which are radiofrequency shielded. The separation of the OR from the DR enabled the individual use of each apparatus. For intraoperative scanning, the magnet was transferred to the OR through the connection door using a track system. To make the awake surgery less challenging in the movable MRI environment, we introduced the minimal draping technique (Parney et al., 2010) to the IMRIS system (Fig. 1B and C) (Lu et al., 2011), thus giving consideration to the airway management and the asepsis of surgical field during the scan. Before the magnet was brought into the OR, all surgical instruments and tables were moved to the back walls of the room outside the 5-Gauss line. All instruments were counted by two persons (a nurse and an MRI technician) to ensure that no ferromagnetic objects were left within the 5-Gauss line. These details are also explained elsewhere (Leuthardt et al., 2011). When the checklist was completed and verified, the magnet was transferred to the OR. During the scanning, the anesthesiologist monitored the vital signs in the control room.

### 2.4 Experimental paradigm and ai-fMRI acquisitions

Before the magnet was moved into the OR and the patient was fed into the MRI cavity, an eight-channel opened head coils (IMRIS, Winnipeg, Canada), which was designed dedicated for the intraoperative scan, was placed onto the patient's head (Fig. 1B and C). The imaging protocols consisted of a 3D T1-weighted magnetization-prepared rapid-gradient echo (MPRAGE) sequence (acquired through axial plane, TR = 1900 ms; TE = 2.93 ms; flip angle = 9°; matrix size = 256 × 215; slice number = 176; slice thickness = 1 mm; field-of-view (FOV) = 250 × 219 mm; acquisition averages = 1; scanning time = 7 min 49 s) in all of the patients except patient 8, for whom we used a T2-weighted fluid-attenuated inversion recovery (T2-FLAIR) sequence (acquired through axial plane, TR = 9000 ms; TE = 99 ms; TI = 2500 ms; flip angle = 150°; matrix size = 256 × 160; slice number = 66; slice thickness = 2 mm; field-of-view (FOV) = 240 × 214 mm; scanning time = 6 min 54 s). Those structural images were used in task-activation overlapping to provide fine anatomical information. Blood oxygenation level-dependent (BOLD) fMRI data was then acquired using a single-shot echo-planar imaging (EPI) sequence with the following parameters for ten out of the 11 patients: TR = 3000 ms; TE = 30 ms; flip angle = 90°; matrix size = 96 × 96; FOV = 240 × 240 mm; 46 contiguous transversal slices; voxel size = 2.5 × 2.5 × 3 mm^3^; 3 dummy scans. For patient 1, we applied a different set of parameters (TR = 3070 ms; TE = 30 ms; matrix size = 92 × 92; FOV = 192 × 192 mm; slice number = 36; slice thickness/gap = 3/0.75 mm; voxel size = 2.1 × 2.1 × 3.8 mm^3^; 3 dummy scans). Please note that, to examine the effects of different scanning durations on ai-fMRI task activation, the patients underwent different ai-fMRI scanning durations. Specifically, for patients 1, 2 and 5, 60 volumes (3 min) were collected; for patients 7, 8 and 9, 120 volumes (6 min) were collected; and for the rest of the patients, 90 volumes (4 min 30 s) were collected. For detailed patient and experiment information, please see Table 1. Please note that, in an ideal situation, there should be several ai-fMRI scans for an operation: after dural opening (for navigation updating) and during tumor resection (for evaluation of the relationship between residue tumor and eloquent areas).

During the ai-fMRI scanning, the patients were asked to perform a simultaneously bilateral fist clenching task in a self-controlled frequency. The task paradigm was a blocked design, with alternating “task – rest – task – rest –…” blocks. Each type of block consisted of 30 s (i.e., the same time for task and rest periods). The total duration of the task experiment varied across patients because of the different lengths of BOLD-EPI acquisitions (i.e., 3, 4.5 or 6 min). To effectively and timely transfer the cue signal to the subjects, we adopted a simple and easily manipulated approach, described as follows. First, the MR technician (Z Yang) sent out the “task” and “rest” signals by switching the OR's light on or off according to the experimental timing, as shown by the Siemens MAGNETOM Verio MRI system. Such signals were received by a neurosurgeon (J.F. Lu) standing by the magnet beside the patients, and the neurosurgeon immediately gave the patients an auditory cue to “perform bilateral hand clenching” or “have a rest”. The neurosurgeon also observed the performance of the patients and made sure that they had successfully completed the task.

### 2.5 ai-fMRI data preprocessing

We employed the Statistical Parametric Mapping toolkit (SPM8, http://www.fil.ion.ucl.ac.uk) to preprocess the data and to perform statistical analyses to generate activation maps. Because three dummy scans were obtained before the data acquisition, no EPI data were deleted before preprocessing. First, for each patient, we reoriented the structure image to a convenient orientation (similar to the orientation in the standard space) by rotation of the image along the *x*, *y*, or *z* axes and applied the rotation parameters to all EPI images of the same patient. This step was performed to compensate for the unusual position of the brain, as all images were acquired in an oblique position, which was suitable for brain surgery. Because the current experiment was a blocked design and the timing shift was quite small when compared with the block length, we did not perform a slice-timing correction. Second, the EPI volumes for each subject were corrected for head motion during scanning using six-parameter rigid transformation, and the motion-corrected EPI images were then resliced. Subjects with a head movement of more than 1 mm in translation or more than 1° in rotation through the *x*, *y*, or *z* axes were excluded from further analyses. Third, each subject's re-oriented structure image was co-registered to her/his averaged EPI images to assign anatomical information to the functional foci. Fourth, all EPI images were spatially smoothed. Please note, several papers have indicated that spatial smoothing is not required for precise functional mapping (Kekhia et al., 2011; Liu et al., 2009), but other papers described the use of a large smoothing kernel (Kokkonen et al., 2009). We performed spatial smoothing to increase the signal-to-noise (SNR) ratio and to enhance the “blob” appearance of active brain regions. In addition, to evaluate which parameter was optimized, different smoothing kernels (no smoothing, or smoothing by using a Gaussian isotropic kernel with full-width at the half maximum [FWHM] of 2, 3 or 4 mm) were applied.

### 2.6 Statistical analyses

Statistical analysis at the individual level for each subject was carried out in the framework of a generalized linear model (GLM), with a task reference (i.e., boxcar time course) convoluted with the canonical hemodynamic response function in SPM8 as the regressor of interest. Except in modeling the task-related BOLD response, we also had several nuisance regressors in the GLM including head motion parameters and extremely low frequency fluctuations (modeled by a set of discrete cosine functions with periods larger than 128 s). Because the use of head motion parameters as covariates is still under debate, we tried different options: 1) including all six head motion parameters as regressors, 2) including those with no significant correlations (*p* > 0.05) with task-related boxcar, and 3) not including any head motion parameters. The task activation map for each subject was generated using a random-effect one-sample *t* test. We did not apply a uniform height threshold to all subjects because in our experience, the task performance, imaging quality, and the tumor's WHO grade and location show high individual variability; therefore, setting the same threshold for all subjects is unreasonable and unnecessary. A strategy was thus adopted to define subject-specific thresholds. First, a very stringent threshold was applied to the task activation *t*-map, in which case no activated cluster existed. Then, we gradually reduced the *t* threshold, allowing motor related areas to show up one by one. The *t* threshold was further reduced, and such a procedure was finally stopped to make sure all task-involved regions were activated while keeping as little “activation” area outside of the regions of interest as possible. The lower limit of the *t* threshold was 1.67 (*p* < 0.05, uncorrected). The *t* threshold could not be reduced below this limit. No extension threshold was applied to the final *t*-maps.

**Fig. 2:**
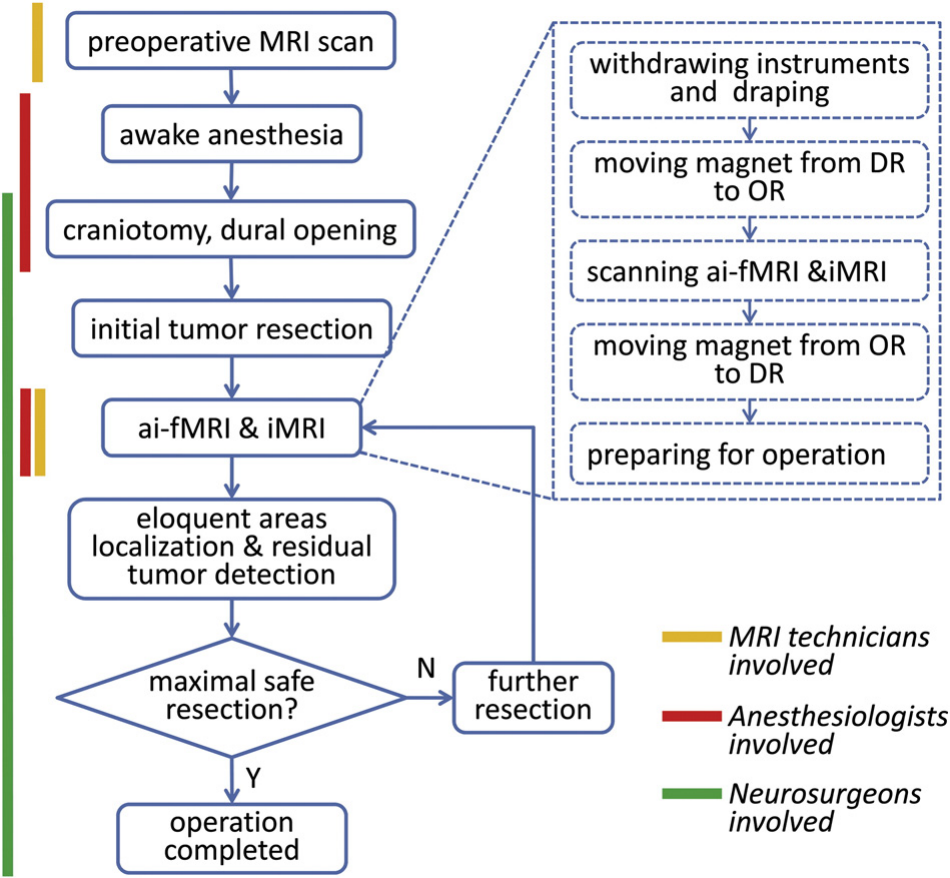
*Workflow of a typical ai-fMRI-guided operation for tumor resection with involved personnel indicated at the left side.* Yellow line represents the procedures that require MRI technicians to participate in; red line indicated that anesthesiologists are involved; and green line for the neurosurgeons.

For a clear demonstration of the whole experimental and data process procedures, please see Fig. 2.

### 2.7 ISM validation

The ai-fMRI was validated by the ISM in two patients (patients 7 and 11) with gliomas in close proximity to motor areas. For ISM, a monophasic square-wave pulse was delivered at 60 Hz through a 5 mm-wide bipolar electrode. The stimulation current ranged from 2 mA to 6 mA. While ISM was in progress, a 4- or 6-contact strip subdural electrode was used to record the afterdischarge activity. The presence of afterdischarge potentials indicated that the stimulation current was too high and would be decreased by 0.5–1 mA as the threshold. The cortical sites of hand motion were identified when the electrodes of the target muscles in the hands recorded compound muscle action potentials (cMAPs) or when the patients complained of passive movements of the hand. Then, the positive sites were marked on the surface of the cortex with sterile tags. After removing part of the tumor, the intraoperative MRI data including ai-fMRI was collected to update the neuronavigational image datasets. The coordinates of positive ISM sites were recalibrated and recorded by the updated image guidance and compared with the ai-fMRI data.

## 3 Results

The locations and pathology of the tumors and the extent of resection in all 11 patients were shown in Table 1. Of the 11 patients, six successfully accomplished the tasks during surgery. Two (patients 9 and 11) could not move their contralateral hands (i.e., the involved hand) as intensely and strongly as their ipsilateral hands. Three (patients 5, 6 and 10, removed from further analyses) could not move their contralateral hands during the scanning, probably because of the sedation level, or the transient heat-shock of brain tissues caused by the bipolar.

**Fig. 3:**
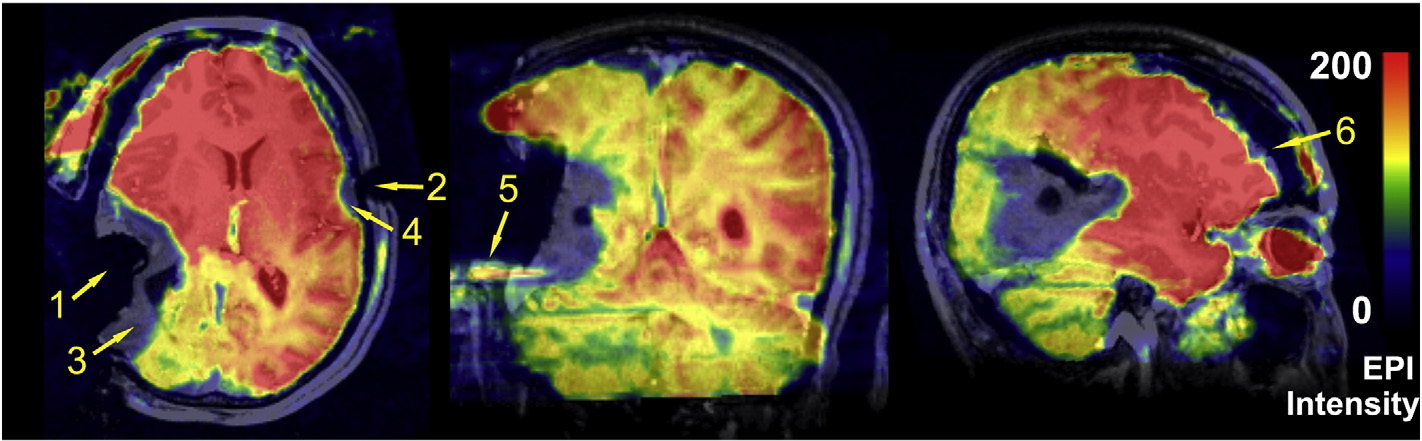
*Demonstration of typical ai-fMRI artifacts.* The artifacts in one of the volumes of intraoperative BOLD-fMRI in patient 6 (this patient was removed from further analyses). The functional image is overlaid on that patient's structural image. Susceptibility artifacts (3, 4) were caused by the resection cavity (1) and the MRI-compatible head fixation pin (2), respectively. Label 5 indicates the Nyquist ghost artifact. Label 6 indicates the brain shift.

**Fig. 4:**
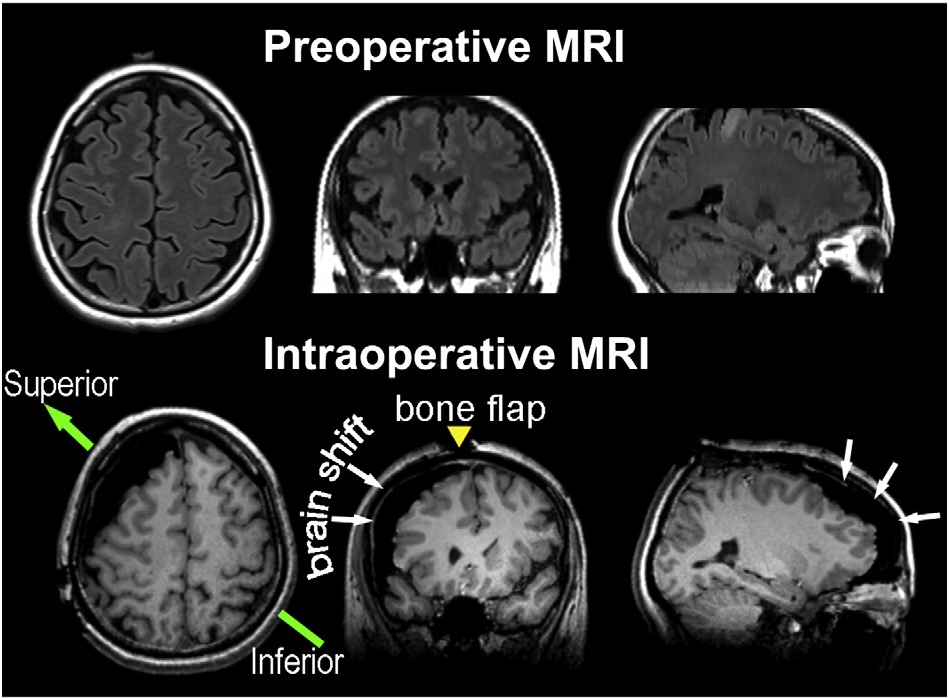
*Demonstration of brain shift caused by craniotomy.* The brain shift was shown using the example of patient 7. The upper row shows the orthographic views of the preoperative structural image (T2-FLAIR). The lower row shows the corresponding planes of the same patient's intraoperatively acquired images (3D-T1-MPRAGE). White arrows show the brain deformation. The yellow arrow indicates the incision line on the scalp (bone flap). The green arrow indicates the direction of the patient's head on the operating table.

During data preprocessing, four out of the 11 patients (patients 5, 6, 9 and 10) were excluded due to excessive head motion (all four patients); serious EPI image artifacts most likely caused by motion or outside radiofrequency pollution (patients 6, 9 and 10, see Fig. 3, label 5); and serious EPI distortion caused by susceptibility changes after resection (patients 5 and 6, see Fig. 3, labels 3 and 4). The data from the other seven patients didn't exhibit significant susceptibility artifacts caused by the head fixation pins (Fig. 3, label 2) or the resection cavity (Fig. 2, label 1), although we did not fill the resection cavity with saline. However, those patients showed significant brain deformation (see the exemplary case of patient 7 in Fig. 4). To quantitatively evaluate the extension of the brain shift, we compared the pre- and intraoperatively acquired anatomical MR images from the same patients and manually measured the maximum brain tissue displacement. For the remaining seven patients, the average displacement distance was 15.5 ± 5.0 mm (ranging from 6.4 to 20.2 mm).

**Fig. 5:**
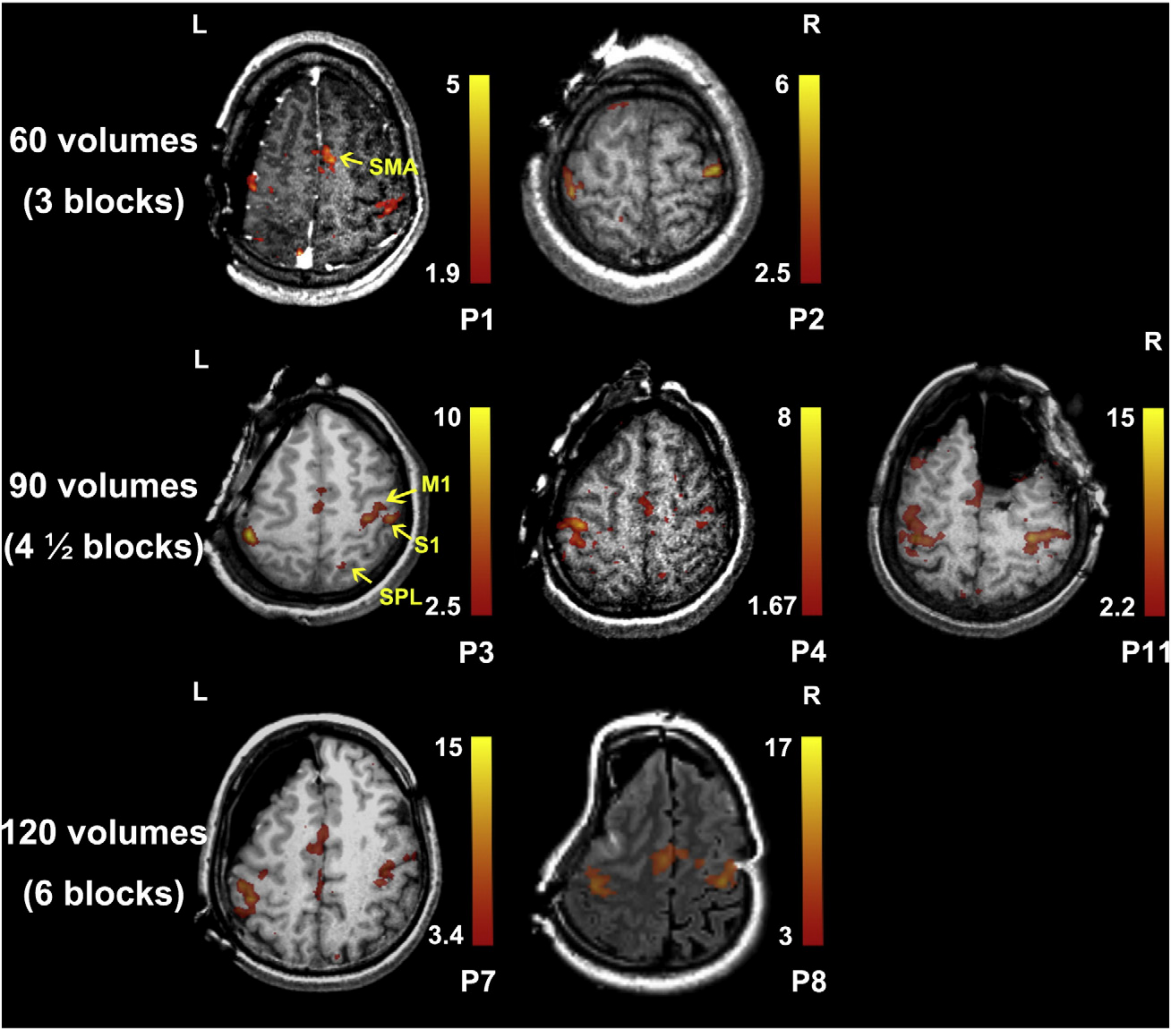
*The ai-fMRI-derived activation maps for all seven patients.* The activation maps (*t*-maps) were superimposed on the individual structural images (T1 or T2-FLAIR). The color bar at the right side of the representative image indicates the threshold of the *t* value. The bilateral primary sensorimotor areas (S1 and M1) and supplementary motor areas (SMA) were activated in all 7 patients. Please note that we only show one representative plane for task activation. The plane in patient 2 did not show the activations of SMA, which were located in the inferior planes. The superior parietal lobe was also activated in patient 3. The first row shows the activation maps derived from 60 volumes (3 block repetitions). The second row shows those derived from 90 volumes (4 and 1/2 repetitions). The bottom row shows those derived from 120 volumes (6 repetitions). P1–11 indicates patients 1–11.

In the remaining 7 patients, weaker task compliance was observed for the left than right hand in patient 11 (details shown in Illustrative cases). In all 7 patients, the activations in sensorimotor cortices were identified by ai-fMRI with the subject-specified threshold defined as described in Statistical analyses. As shown in Fig. 5, the activations were mainly located in bilateral primary motor or sensory cortices (M1, S1) and the supplementary motor areas (SMA). For some patients, the activations also extended to the superior parietal cortex (SPL) and the premotor cortex. In two cases (patients 7 and 11), the activations of the motor cortex were validated with the ISM (marked by “H” labels). The neuronavigational system using both the updated i-sMRI and the updated ai-fMRI confirmed that all ISM positive sites (one “H” label for patient 7 and two “H” labels for patient 11) were located within the ai-fMRI-derived hand motion blobs (for more details, please see Illustrative cases). After operations, both of them suffered from transient postoperative motor deficits (could be caused by the cerebral edema or SMA syndrome). However, no permanent motor deficits were found.

By comparing the results with different smoothing kernels (non-smooth, isotropic FWHM of 2 mm, 3 mm, and 4 mm), we found that there were small distinctions among the results with smoothing kernels equal to or larger than 2 mm (with a slightly larger spatial extension for the larger smoothing kernel; however, due to small sample size, the statistical relationship between SNR and smoothing kernel size was not evaluated here). The extent of activations (i.e., cluster size) and the SNR (i.e., as roughly evaluated by the extension of false positive areas located outside of the sensorimotor cortices) were largely increased with the FWHM kernel of 2 mm compared to those without smoothing (see an example case in Supplementary Fig. 1). Therefore, we preliminarily concluded that spatial smoothing increased SNR and detected eloquent areas with higher sensitivity and specificity. However, quantitative analysis should be performed using a larger sample size. In addition, an investigation on how to include head motion parameters as nuisance regressors was also carried out. Here, we compared the peak *t* values in both sides of the sensorimotor areas (SM1) (i.e., local maxima) and the peak *t* value across the whole brain (global maxima) among the three cases of head-motion inclusion (see Statistical analyses). However, we did not find a clear difference between “no head-motion parameter inclusion” and “including all head-motion parameters”. But for the patients with task-design-related head motion (patients 3, 7 and 8), “including all head-motion parameters” tended to produce the most suboptimal results (for more details, please see Discussion and Supplementary Table 1). Moreover, while using the same threshold (*p* < 0.05, uncorrected), we found that more ai-fMRI acquisition numbers (i.e., time points) produced a larger extension of task activation and a higher peak intensity. Specifically, using 60 volumes (3 task block repetitions), we obtained a peak *t* of 3.48–5.64 in bilateral SM1 when not including head-motion parameters as covariates; using 90 volumes, the peak *t* was 7.23–15.48, and using 120 volumes, the peak *t* was 15.09–18.85 (see Discussion for comprehensive evaluation).

### 3.1 Illustrative cases

#### 3.1.1 Patient 7

**Fig. 6:**
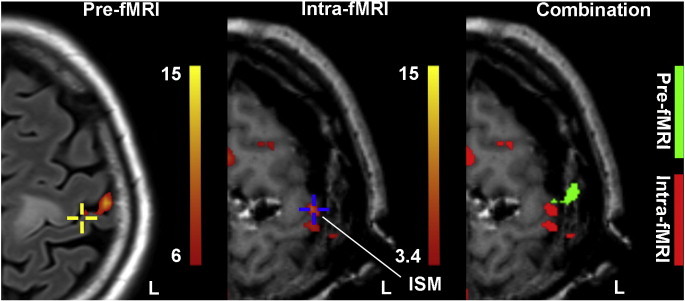
*Comparison between pre-fMRI and ai-fMRI-derived activation results in patient 7.* The preoperative (T2-FLAIR, the underlay image in the left panel) and intraoperative (T1-MPRAGE, the underlay image in the middle and right panels) structural images were co-registered, allowing the pre- (in green) and ai-fMRI-derived activation maps (in red) to overlapped in the right panel. The blue cross indicates the ISM positive site of hand motion, which was recorded using the updated neuronavigation. The same location of the ISM positive site in the middle panel is marked in the left image with a yellow cross. Due to the brain shift, the ISM-positive site that was located inside of the ai-fMRI activation blobs moved outside of the pre-fMRI activations.

After a first generalized seizure, this 51-year-old female patient underwent cerebral MRI that showed a lesion in the left precentral gyrus. The pre-fMRI and the structural MRI were acquired the day before surgery. In addition, awake surgery and ISM were also performed to preserve the motor function. For the acquisition of ai-fMRI, the paradigm had six block repetitions. The ISM-positive site of hand motion was recorded by updated navigation (Fig. 6, shown by a blue cross in the middle image). To assess the brain-shift effect on the localization of hand motion, we compared the location of activation areas derived from preoperative and those from ai-fMRI. To achieve this, the intraoperative structural MRI (T1-MPRAGE) was co-registered with the preoperative structural MRI (T2-FLAIR) using a rigid body transformation in SPM8 to make the two activation maps in the same space. By overlapping the pre- and ai-fMRI results, we observed a significant shift of activation blobs induced by the surgical procedure (the right image of Fig. 6) and that the ISM-positive site was located outside of the areas that were defined by pre-fMRI (the left image in Fig. 6, with the yellow cross showing the corresponding location of the blue cross in the middle image). If brain surgery was guided by the un-updated fMRI datasets (i.e., the preoperatively acquired datasets only) rather than by the updated ai-fMRI information, the eloquent areas could be damaged in extensive resection. Actually, the muscle strength of right limbs was decreased for about 2 weeks, but it was restored at the 3-month follow up.

#### 3.1.2 Patient 11

**Fig. 7:**
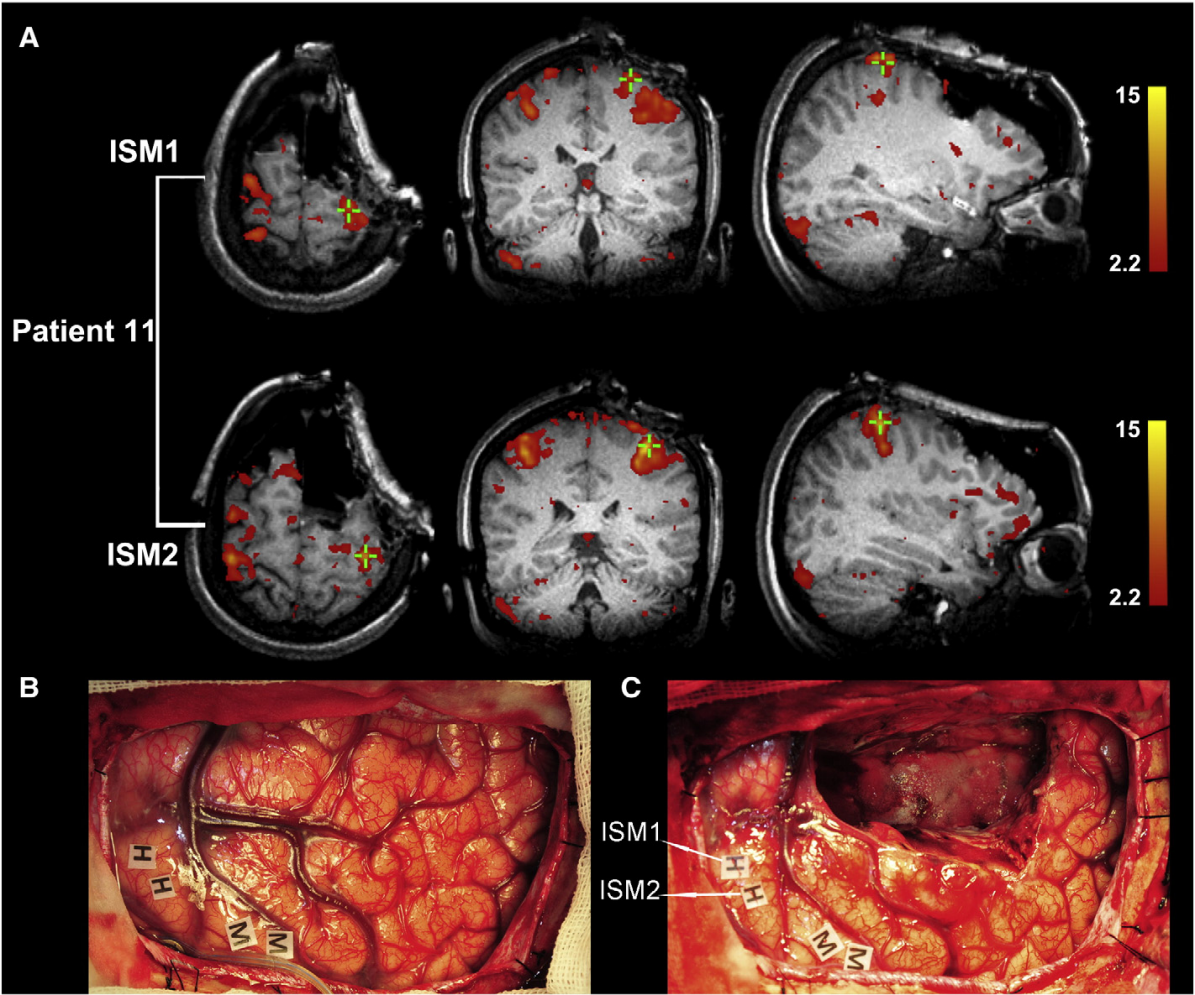
*Validation by intraoperative stimulation mapping (ISM) in patient 11.* Two positive sites (ISM1 and ISM2, indicated by green crosses) were identified and are displayed (A). The intraoperative photographs are also shown (the anterior part is right, the back is left), including one taken before tumor resection (B) and the other taken after subtotal resection (C). Awake mapping allowed for the detection of the primary motor cortex of the hand (H) and face (M). Note that the images in A and the photograph in C were taken during the same time period.

This patient was a 39-year-old woman with repeated seizure attacks prior to admission. MRI indicated a low grade glioma in the right frontal lobe. Awake surgery was performed to protect motor function. Brain stimulation mapping identified the hand and mouth motion sites. After debulking the tumor, the intraoperative MRI was taken to obtain the updated structural and functional images for the detection of the residual and functional areas. The ai-fMRI successfully localized the motor cortex through both offline (see Materials and methods) and online analyses (using our imaging workstation, for more details on the online analyses for all 7 patients please see *Supplementary materials*). As demonstrated in Fig. 7, both of the ISM-positive sites (ISM1 and 2, as shown by two “H” labels in Fig. 7B and C) for hand motion were located within the activation areas in the right sensorimotor cortex. Because i-sMRI had confirmed that there was residual tumor tissue, we planned to perform extensive resection. After updating both the structural and functional information in the neuronavigator, we assessed the real-time relationship between the border of the cavity and the ai-fMRI-derived activation clusters and confirmed that the resection border was far from the eloquent area (larger than the safety distance). The ai-fMRI result was further validated by “golden standard” ISM. Thus, we did the further resection. At last, the tumor was extensively removed. One month after operation, the patient exhibited a transient motor deficit of the left upper limb (i.e., SMA syndrome), but she fully recovered to normal muscle strength at the 3-month follow-up.

## 4 Discussion

In this study, we administered a canonical hand motion task to test the feasibility and reliability of i-fMRI in identifying sensorimotor areas during awake surgery. For all seven patients involved, the activations derived from ai-fMRI were all located at the regions of interest; for two patients with ISM data, the i-fMRI findings were in concordance with the golden standard.

Few literatures have reported the implementation of i-fMRI, although intraoperative MRI has attracted increasing interests over the past decade due to its ability to detect tumor remnants and compensate for brain shift (Kuhnt et al., 2011; Lang et al., 2011; Lu et al., 2011; Nimsky et al., 2004a; Senft et al., 2011; Sun et al., 2011; Wu et al., 2009). The concept of i-fMRI was introduced in 1998 in two healthy volunteers using a low field-strength (0.5 T) MR scanner (Gering and Weber, 1998); following that study, several reports also performed similar attempts using low field-strength MR scanners (Azmi et al., 2007; Schulder et al., 2003). In addition, several anesthetized patients have been reported in clinical practice with high field-strength (1.5 T) MR (Gasser et al., 2005). Although i-fMRI has demonstrated the potential to detect various cortical regions, the “real” applications of i-fMRI have been progressing slowly. This may be due to three specific reasons: first, a low field strength magnet cannot fulfill the requirement for high spatial and temporal resolution nor can it achieve a contrast-to-noise ratio that is favorable in “true” intraoperative mapping; second, it is impossible to activate a multiple functional system while patients are anesthetized; and third, the anesthetic drug may cause an altered hemodynamic response that results in false positive or false negative in the detection of activation (Gasser et al., 2005).

In the current study, we have successively demonstrated the feasibility of i-fMRI during awake surgery using a high field strength (3.0 T) MR system. By integrating experimental design with the volitional execution of a task, while also avoiding an effect from anesthesia, ai-fMRI has the potential to fully explore the capacity of fMRI on functional mapping. In the future, other experimental tasks can be straightforwardly applied for mapping higher or more complex cognitive systems. For example, we can apply a subject-specified task, e.g., a verb generation task, to identify areas associated with speech production in cases with tumors located near the language areas. We propose that, using the subject-specified protocol and ai-fMRI one can map the eloquent area more freely with higher sensitivity and specificity.

### 4.1 Technical experiences

Here, we would like to share our experience with ai-fMRI considering aspects of 1) awake surgery, 2) data acquisition and 3) data analyses.

#### 4.1.1 Awake surgery

Awake surgery was originally introduced for the surgical treatment of epilepsy, and it has subsequently been used in patients undergoing surgeries for cerebral tumors and other lesions near critical brain areas for brain mapping (Duffau, 2011; Hamberger, 2007; Ojemann et al., 1989; Piccioni and Fanzio, 2008). This study is the first application of awake surgery to i-fMRI. To achieve ai-fMRI safely and successfully, several technical aspects should be considered. First, in our experience, attention to detail in terms of patient selection and training is critical to the success of this procedure. Analogous to the ISM, the patient's complete cooperation and participation determines the task performance (Goebel et al., 2010; Piccioni and Fanzio, 2008). Hence, preoperative evaluation is necessary for the anesthesiologists to evaluate the patient's health, cooperation, and airway characteristics (Bonhomme et al., 2009; Piccioni and Fanzio, 2008). Moreover, the patients should be trained by the neurosurgeons to perform the intraoperative tasks before surgery. The patients who are unsuitable for awake surgery should be excluded. Second, a combination of MAC protocol and minimal draping is the optimized solution for ai-fMRI acquisition. During the acquisition of ai-fMRI, the biggest challenge is to keep patients awake as well as ensure that the sterility of the operative field is maintained. The method of combining awake surgery and intraoperative MRI proposed by Leuthardt et al. (2011) was unable to keep the patients awake during the MRI scan because the patients were wrapped in a full-body drape for image acquisition using a laryngeal mask to maintain a safe airway. Third, intraoperative monitoring is necessary, particularly during the acquisition of ai-fMRI. The anesthesiologist should monitor the vital signs, and one of neurosurgeons should remain beside the patients to observe the performance and to watch for adverse events, although no severe adverse events were recorded during awake surgery and ai-fMRI acquisition in this study. None of the 11 patients had any complaints, including MRI noise, during the postoperative interview.

#### 4.1.2 Data acquisitions

Intraoperative functional imaging poses a great challenge with regard to possible image distortion caused by the susceptibility of head fixation pins, intracranial air, the restricted choice of MR head coils, and the OR environment (Gasser et al., 2005, 2011; Ulmer et al., 2009, 2010). First, although the two-piece designed open coils in the intraoperative MRI provided high-quality images for the structural image, in our preliminary experience, the functional images generated by intraoperative MRI were not as excellent as those obtained with a conventional closed-type coil. To widely apply ai-fMRI in clinical applications in the future, the imaging quality of ai-fMRI and its effect on functional mapping should be fully assessed. Second, the OR environment also had an important impact on the image quality (i.e., SNR), despite the fact that all the instruments were moved away from 5-Gauss line. The hampered image quality may have been due to the probable existence of radiofrequency leakage from outside equipment, such as an incompatible anesthesia pump and a constant temperature cabinet. Third, the compatible titanium skull pins and resection cavity caused superficial susceptibility artifacts in some cases. To minimize their influence, it is recommended that the pins should be located far from the activation site.

The number of data acquisitions is also an opened issue. Generally, more temporal acquisitions will increase statistical power and lead to higher sensitivity and specificity in activation detection. However, prolonged scanning time will cause problems to the patient and increase the rate of infection. Therefore, optimization of the ai-fMRI scanning time is necessary. We had obtained data with different sample sizes from different subjects (3, 4.5 and 6 block repetitions); therefore, we can qualitatively evaluate the optimized repetition number. Our results demonstrated that results from 4.5 blocks or longer were better than those from 3 blocks. This indicated that at least 4–5 blocks should be included for task design using ai-fMRI, although 3 blocks seemed to be adequate for sensorimotor mapping. Please note that such an evaluation could be affected by variations in the task performance, scanning environment, MR machine quality, task design or the involved functional systems. Therefore, in the future, a more dedicated study (i.e., within-subject comparisons in cases of various scanning time) should be carried out to define the optimized scanning time. In addition, different task paradigms should be tested to define the optimized task design for each functional system.

#### 4.1.3 Data processing

Because the data from ai-fMRI is quite different from that of conventional fMRI, several methodological issues in data processing should be discussed, including smoothing kernels, nuisance regressors, and analyzing speed. First, a smoothing kernel is important for eloquent area localization. Slight smoothing will identify more spatially localized activations; however, it is accompanied by a reduced SNR. Comparisons revealed that the results of smoothing with different FWHM kernels outperformed those without smoothing. We found that, for the data with smaller acquisition numbers (patients 1 and 2), smoothing increased the sensitivity in the identification of the functional areas (results not shown). However, a large smooth kernel (≧ 4 mm) is not recommended for the processing of ai-fMRI because smoothing may artificially combine activations from adjacent but functionally and/or anatomically distinct brain regions, and that activation from large draining vessels may be smoothed into neighboring neuronal tissue (Fransson et al., 2002; Geissler et al., 2005).

Second, nuisance regressors in GLM are always under debate. Including head motion parameters as covariates will change the activation result, but the extent to which the result can be altered is unknown. Because mapping eloquent areas requires accuracy, such a problem gains significance. We assessed the effect of motion parameters as nuisance covariates on the peak *t* values of the whole brain and bilateral sensorimotor areas (see Supplementary Table 1 for details). We have hypothesized that including motion parameters with significant correlations to the task paradigm would decrease peak intensity and cluster extension. The results showed no clear differences between “no inclusion of motion parameters (NO)” and “including all of them (ALL)”. However, for subjects with task-related head motion, “partial inclusion (only for those with no significant correlation to task design, PI)” as well as NO were more optimal than ALL. Therefore, we cannot provide suggestions for future studies about whether to include head motion parameters, but we would like to suggest that if the head motions are correlated with the task design, one should not include them as nuisance regressors. These findings require validation from further studies with more patients.

Third, we have shown the possibility of online ai-fMRI data analysis using the Siemens workstation (Syngo Multi Modality Workplace, Siemens AG, Erlangen, Germany). The description of the procedure and the results can be found in *Supplementary materials*. By comparing the activation patterns between online and offline analyses, the extent and location of sensorimotor areas were similar but slightly smaller in five patients, excluding the first two patients (patients 1 and 2). The failure of online analysis in these two patients may have been caused by the use of non-smoothed data together with a relatively smaller fMRI acquisition number. When a sufficient number of volumes with appropriate spatial smoothing are achieved, online analysis could provide a result that is as favorable as that from offline analysis. Using online analyses has other benefits: it is more convenient for the neurosurgeons, and it can be processed faster than offline analysis and is thus more suitable for updating information during brain surgery. Perhaps in the future, online analysis will be performed instead of offline analysis, unless significant methodological advances in offline analysis are developed.

### 4.2 Future works

There are several issues that need to be addressed in the future. First, in the current study, we simply selected and analyzed the data from seven patients. A larger number of patients with consistent tumor locations should be involved in the future. Second, due to the time constraints, we only obtained ai-fMRI after partially removing the tumors. We suggested that the ai-fMRI data should be acquired at a minimum of two stages during the operations (after dural opening and after resection). It would be more meaningful to acquire ai-fMRI right behind a dural opening because it could guide the resection at the very start of the procedure. Third, the processing pipeline should be optimized and standardized. It is required to develop a dedicated real-time data reconstruction, noise removal and activation detection methods to get the task activation blobs in a real-time way. Another direction is to develop a functional localization-dedicated online data processing pipeline rather than relying on the oversimplified one that is provided by the workstation of the fMRI manufacturer, and to make sure that when the ai-fMRI scan is over, the activation map will come out in seconds. Provided with a modern high-speed computer, it is easy to both shorten the computing time and adopt a sophisticated data processing method. Finally, other higher-order functional systems should be investigated using ai-fMRI, which offers distinct advantages over ISM.

## 5 Conclusions

This is the first study to reveal ai-fMRI for the localization of sensorimotor areas in awake patients. Our findings confirmed that ai-fMRI is feasible and reliable in identifying functional areas during awake craniotomy. Some practical issues on performing the ai-fMRI on awake patients were systemically discussed and guidelines were provided.

## Figures and Tables

**Table 1:** Demographic, clinical and experimental information for all of the involved brain tumor patients.

Patient data						Data acquisition of ai-fMRI		Dose of awake anesthesia	
Patient no.	Sex/age(y)	Tumor location	Pathology	WHO grade	Extent of resection^a^	Intraoperative performance^b^	Blocks	Propofol^c^	Remif-Entanil^d^
1	M/47	L temporal, parietal	Anaplastic oligodendroglioma	III	Total	Good	3	1.0	0.015
2	M/31	L frontal, insula	Astrocytoma	II	Subtotal	Good	3	1.6	0.020
3	F/40	L frontal, insula	Astrocytoma	II	Subtotal	Good	4.5	1.7	0.020
4	F/48	L insula	Astrocytoma	II	Total	Good	4.5	1.7	0.015
5	M/27	L insula	Astrocytoma	II	Subtotal	Failed (right hand)	3	1.0	0.030
6	M/58	L temporal, parietal	Glioblastoma	IV	Total	Failed (right hand)	4.5	0.8	0.020
7	F/51	L precentral gyri	Astrocytoma	II	Total	Good	6	1.3	0.015
8	M/31	L frontal, insula	Anaplastic astrocytoma	III	Subtotal	Good	6	1.3	0.020
9	M/26	L frontal, insula	Glioblastoma	IV	Total	Weaker (right hand)	6	1.0	0.020
10	M/44	L frontal	Astrocytoma	II	Total	Failed (right hand)	4.5	1.6	0.015
11	F/39	R frontal	Astrocytoma	II	Total	Weaker (left hand)	4.5	1.5	0.015

ai-fMRI = “awake” intraoperative functional MRI; WHO = World Health Organization.

a The extent of resection was determined by volumetric analysis. Total was defined as 100% resection of the tumor volume based on T2-FLAIR or T1 contrast. Subtotal was defined as greater than 90%.

b Good means the bilateral hands normally and equally moved. Failed (with the failed side) means that the hand indicated in parenthesis could not move. Weaker (with the weaker side) means that the hand indicated in parenthesis could not move as intensely and strongly as the other hand.

c Propofol was administered using a target-controlled infusion (TCI) technique. The effect-site concentrations (μg ml^− 1^) before discontinuation for ai-fMRI were shown in this column.

d The maintenance doses of remifentanil (μg kg^− 1^ min^− 1^) during ai-fMRI scan.
