# Supplementary material for: “Awake” intraoperative functional MRI (ai-fMRI) for mapping the eloquent cortex: Is it possible in awake craniotomy?
Source: Neuroimage Clin. 2012 Dec 12;2:132–42. doi: 10.1016/j.nicl.2012.12.002 (PMC3777788; doi:10.1016/j.nicl.2012.12.002)
Supplement: Supplementary file 1 — Supplementary materials. [file mmc1.doc]

**Supplementary** **Materials**

**Online data analyses**

We also detected task activation with the help of a commercially available post-processing workstation (Syngo Multi Modality Workplace, Siemens AG, Erlangen, Germany). Immediately after the functional data acquisition, the *t*-maps were automatically generated using the following processing procedures: 1) the raw ai-fMRI data were corrected for head motion; 2) the data were then fed into a general linear model with a task-related boxcar convoluted with canonical HRF as the regressor of interest and with a transition-state-related effect and an extremely low frequency trend (with period larger than 120 s), as modeled by discrete cosine functions as nuisance regressors. No spatial smoothing (i.e., spatial filtering) was performed in the preprocessing step, and no head motion parameters were included as covariates; then, 3) *t*-maps were generated. To achieve better visualization, we adopted the Neuro 3D task card contained in our imaging workstation (Syngo Multi Modality Workplace, Siemens AG, Erlangen, Germany) to overlay the task activation map onto the intraoperatively acquired anatomical data. To make a comparison between online- and offline-derived activation results, the same threshold used for the offline data processing was applied to online-derived result. We also demonstrated the corresponding planes on the offline result (see Fig. 4 in the main article) for the online-derived *t-*maps for all 7 patients (Supplementary Fig. 2). In our practice, it usually took approximately 1-2 min to prepare the final result for visualization using online analyses, and it took an additional 5 min to register these results to the neuronavigational systems. No obvious patterns of sensorimotor activations were identified bilaterally in patients 1 and 2 (P1 and P2) or in the right hemisphere of patient 4 (P4). In comparing these results with those from the offline analyses, the extents of the activation areas after the online analyses were slightly smaller in patients 3, 4, 7, 8 and 11.


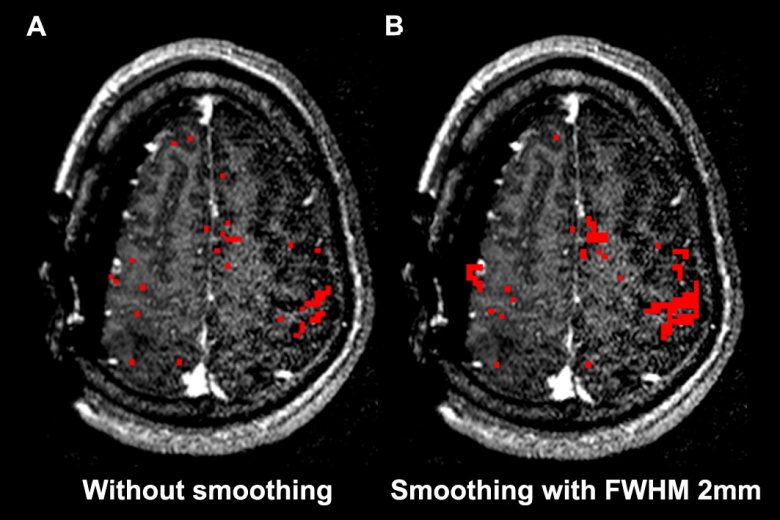


**Supplementary Fig. 1. Comparison of the task activation result between no smoothing and smoothing with a Gaussian isotropic kernel with full-width at the half maximum of 2 mm in an exemple patient (patient 1).** The thresholds of the *t* values for both activation maps were 2.5. To clearly demonstrate the effect of spatial smoothing on the final activation map, the overlay images were not up-sampled using an interpolation approach. We could observed the formation of larger blobs and less false activation (i.e., “activation” detected outside of the motor cortices) when smoothing was adopted to preprocess the ai-fMRI data.


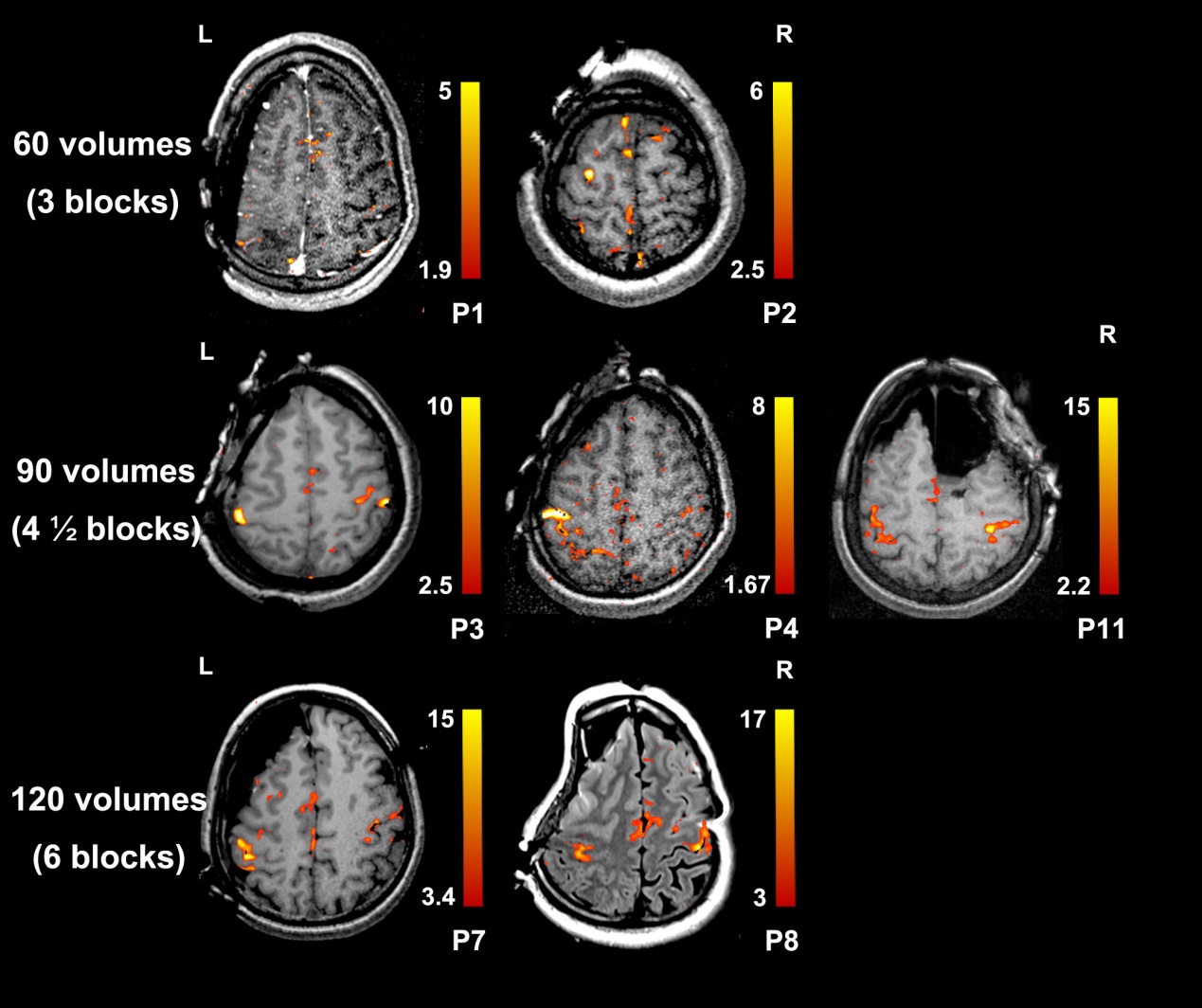


**Supplementary Fig. 2. The representative activation maps superimposed on the individual structural images (at the same planes as Fig. 4) in the 7 patients using online analyses.** The right color bar indicates the threshold of the *t* values, which was kept the same as that in the offline analyses.

**Supplementary Table 1** Peak *t* values in both sides of the sensorimotor areas and across the whole brain in the three cases of head motion regressor inclusion (see *Statistical Analyses* in the main article, which describes the different considerations for head motion)

| Patients No. | Without head-motion regressors | | | With head-motion regressors | | | With selected head-motion regressors ^a^ | | |
| --- | --- | --- | --- | --- | --- | --- | --- | --- | --- |
|  | Whole ^b^ | Left SM1^c^ | Right SM1 | Whole | Left SM1 | Right SM1 | Whole | Left SM1 | Right SM1 |
| 1 | 5.64 | 3.48 | 5.64 | 5.40 | 3.64 | 5.23 | NA | NA | NA |
| 2 | 6.45 | 5.45 | 5.50 | 7.26 | 7.26 | 6.55 | NA | NA | NA |
| 3 | 11.19 | 10.57 | 11.19 | 10.47 | 9.71 | 10.47 | 11.30 | 11.04 | 11.30 |
| 4 | 11.57 | 9.77 | 7.23 | 8.30 | 6.63 | 5.27 | NA | NA | NA |
| 7 | 17.76 | 17.76 | 15.54 | 13.33 | 13.33 | 10.19 | 15.78 | 15.78 | 12.81 |
| 8 | 18.85 | 15.09 | 18.85 | 18.00 | 15.48 | 18.00 | 19.23 | 17.03 | 19.23 |
| 11 | 15.48 | 13.22 | 15.48 | 14.59 | 11.98 | 14.59 | NA | NA | NA |

^a^ Using head-motion parameters that showed no significant correlations (*p* > 0.05) with the task-related boxcar time course;

^b^ The peak *t* value across the whole brain (i.e., global maxima);

^c^ The peak *t* value within the left side primary sensorimotor area (SM1), that is, local maxima;

NA: not available
